# From Forest Soil to the Canopy: Increased Habitat Diversity Does Not Increase Species Richness of Cercozoa and Oomycota in Tree Canopies

**DOI:** 10.3389/fmicb.2020.592189

**Published:** 2020-12-22

**Authors:** Robin-Tobias Jauss, Susanne Walden, Anna Maria Fiore-Donno, Kenneth Dumack, Stefan Schaffer, Ronny Wolf, Martin Schlegel, Michael Bonkowski

**Affiliations:** ^1^Molecular Evolution and Animal Systematics, Institute of Biology, University of Leipzig, Leipzig, Germany; ^2^Terrestrial Ecology, Institute of Zoology, University of Cologne, Cologne, Germany; ^3^German Centre for Integrative Biodiversity Research (iDiv) Halle Jena Leipzig, Leipzig, Germany

**Keywords:** protists, canopies, metabarcoding, habitat filtering, forest ecosystems, illumina

## Abstract

Tree canopies provide habitats for diverse and until now, still poorly characterized communities of microbial eukaryotes. One of the most general patterns in community ecology is the increase in species richness with increasing habitat diversity. Thus, environmental heterogeneity of tree canopies should be an important factor governing community structure and diversity in this subsystem of forest ecosystems. Nevertheless, it is unknown if similar patterns are reflected at the microbial scale within unicellular eukaryotes (protists). In this study, high-throughput sequencing of two prominent protistan taxa, Cercozoa (Rhizaria) and Oomycota (Stramenopiles), was performed. Group specific primers were used to comprehensively analyze their diversity in various microhabitats of a floodplain forest from the forest floor to the canopy region. Beta diversity indicated highly dissimilar protistan communities in the investigated microhabitats. However, the majority of operational taxonomic units (OTUs) was present in all samples, and therefore differences in beta diversity were mainly related to species performance (i.e., relative abundance). Accordingly, habitat diversity strongly favored distinct protistan taxa in terms of abundance, but due to their almost ubiquitous distribution the effect of species richness on community composition was negligible.

## Introduction

Forest ecosystems harbor 80% of terrestrial biodiversity, influence climate through biogeochemical cycles, and provide ecosystem services to society (Bonan, 2008; Aerts and Honnay, 2011). On a global scale, there are more than 3 trillion trees on Earth, of which 43% can be found in tropical and subtropical regions and 22% in temperate biomes (Crowther et al., 2015). Their tree canopies form the functional interface between 90% of Earth’s terrestrial biomass and the atmosphere (Ozanne et al., 2003; Ellwood and Foster, 2004) and contain a variety of heterogeneous microhabitats conducive to the evolution of epiphytic plants, animals, and microorganisms (Nadkarni, 2001). Furthermore, communities inhabiting tree canopies are considered to contribute significantly to the maintenance of the diversity, resiliency, and functioning of forest ecosystems (Thompson et al., 2009).

Although the coexistence of habitat specialist and generalist species is widely observed, habitat heterogeneity tends to favor generalist species, while specialists benefit from homogeneous habitat conditions (Marvier et al., 2004; Devictor et al., 2008; Verberk et al., 2010). Yet, one of the general patterns in community ecology is the increase in species richness with increasing habitat heterogeneity (MacArthur and MacArthur, 1961; Williams, 1964; Hortal et al., 2009; Stein et al., 2014; Oloo et al., 2016). Accordingly, the presence of microhabitats differing by environmental factors (e.g., UV radiation, temperature, and moisture) within tree canopies was shown to favor biodiversity of a variety of organisms, including epiphytes (Lyons et al., 2000; Nadkarni, 2001), birds (Goetz et al., 2007), small mammals (Carey and Wilson, 2001), and arthropods (Hijii et al., 2001; Ishii et al., 2004). Similarly, on a smaller, microbial scale, tree-colonizing microorganisms (e.g., Bacteria, Archaea, and microfungi) formed highly specific communities across broader microhabitat classes (soil, stems, and leaves; Cregger et al., 2018). In addition, it was shown that different plant species harbor species specific leaf-associated bacterial communities (Lambais et al., 2006; Vorholt, 2012) and even the cryptogamic epiphytes (bryophytes, macrolichens) on trees were shown to harbor highly adapted bacterial communities (Aschenbrenner et al., 2017). Accordingly, we hypothesized that the diversity of unicellular eukaryotes (protists) would also differ between different microhabitats within tree crowns. So far, molecular studies reported distinct protistan communities in mosses (Mitchell et al., 2004; Mieczan and Tarkowska-Kukuryk, 2014), lichens (Bates et al., 2012; Mazei et al., 2016), phytothelmata (Carrias et al., 2001; Dunthorn et al., 2012) as well as root associated communities (Turner et al., 2013; Dumack et al., 2020). Fungal phyllosphere communities determined the community composition of subsequent litter fungal communities (Guerreiro et al., 2018). Correspondingly, a recent study on protistan diversity of tropical forest soils hypothesized that some protists may have originated from tree canopy communities from where they were washed down by rain (Mahé et al., 2017). Nevertheless, a comprehensive comparative assessment of protistan communities of tree canopies across different tree microhabitats and a comparison to forest soil communities is still lacking.

Accordingly, the aim of this study was to shed light on unicellular eukaryotic diversity and community composition in forest soils and the canopy region. Using a metabarcoding approach, we assessed the diversity of two prominent and potentially plant associated taxa of protists (Ploch et al., 2016; Flues et al., 2017; Sapp et al., 2018), namely the Cercozoa (Rhizaria) and the Oomycota (Stramenopila). We sampled specific microhabitat compartments across two vertical levels – forest soils and the canopy region – of three autochthonous tree species in a temperate floodplain forest. To ensure exhaustive coverage of the investigated taxa, taxon-specific primers were used to amplify protistan DNA. Considering variation in taxonomic resolution of DNA barcodes, two different markers were targeted in this study: the hypervariable V4 region of the 18S rRNA gene and the Internal Transcribed Spacer 1 (ITS1) for barcoding cercozoan and oomycete communities, respectively. Following the terminology of Stein and Kreft (2015), we define *environmental heterogeneity* as an “umbrella term for all kinds of spatial heterogeneity, complexity, diversity, structure, or variability in the environment,” while, we are focusing here in particular on the sub-categorical term *habitat diversity* as a measurement of habitat richness, i.e., the number of distinct (micro-)habitats and habitat types.

Unveiling the distribution patterns of Cercozoa and Oomycota will contribute to the understanding of environmental factors shaping protistan communities in forest ecosystems and of tree canopies for microbial biodiversity. We hypothesized (1) to find different microhabitat-specific protistan communities in tree canopies, (2) an increase of species richness with habitat diversity, and (3) forest floor litter communities to reflect the composition of canopy phyllosphere communities.

## Materials and Methods

### Sampling and DNA Extraction

Microhabitat samples were collected in October 2017 in cooperation with the Canopy Crane Facility in the floodplain forest in Leipzig, Germany (51.3657 N, 12.3094 E). We sampled three different specimens of three autochthonous tree species: The small-leaved lime (*Tilia cordata*), the European ash (*Fraxinus excelsior*), and the pedunculate oak (*Quercus robur*). The samples can be classified into two strata: (i) canopy samples and (ii) ground samples. Canopy samples were taken at 20–30 m height with replicates at all four cardinal directions of each tree. We choose *a priori* a number of microhabitats that could be immediately distinguished and easily sampled in the tree crown. The following seven microbial microhabitat compartments related to tree surface were sampled: Fresh leaves, deadwood from dead and dried out branches, bark, arboreal soil and three cryptogam epiphytes (lichen and two moss genera, *Hypnum* and *Orthotrichum*). In addition, two ground samples (soil and leaf litter with four replicates per tree) at 2 m distance from each trunk were sampled. The soils were collected at the surface layer (~10 cm depth after removal of leaf litter and stones) throughout each station. All 324 samples were stored at −22°C until further processing. For DNA extraction, all canopy and litter samples were decorticated and/or chopped with a sterile razor blade and cut into small, regular pieces. DNA extraction was done according to the manufacturer’s instruction with the DNeasy PowerSoil kit (QIAGEN, Hilden, Germany). DNA concentration and quality were checked using a NanoDrop Spectrophotometer (NanoDrop Technologies, Wilmington, United States). For following PCR amplification, all four replicates of each microhabitat per tree were pooled.

### PCR Amplification, Barcoding, and Sequencing

PCRs with taxon specific primers were conducted in two steps. The hypervariable V4 region of the 18S ribosomal RNA gene (SSU rDNA) was used for cercozoan community profiling with specific primers (Fiore-Donno et al., 2020). For the first PCR, the forward primers S616F_Cerco and S616F_Phyt were mixed in the proportions of 50 and 50%, and used with the reverse primer S963R_Phyt. For a following semi-nested PCR, a mixture of the reverse primers S947_Phyt and S947_Vamp in an equal proportion has been used as described in Fiore-Donno et al. (2020). The thermal program for the first and second PCR consisted of an initial denaturation step at 95°C for 2 min, 24 cycles at 95°C for 30 s, 52°C for 30 s, 72°C for 30 s; and a final elongation step at 72°C for 5 min. For amplifying the ITS 1 of the oomycete communities, we used the specific primer pair ITS_177F and 58SR_Oom (Fiore-Donno and Bonkowski, 2020). Amplicons of the first PCR were again used as template for a semi-nested PCR with the primer pair I1786F_Stra and 58SR_Oom. The thermal program for the first and second PCR started with a denaturation step at 95°C for 2 min, followed by 24 cycles at 95°C for 30 s, 58°C for 30 s, 72°C for 30 s; and a final extension step at 72°C for 5 min.

We used 1 μl of DNA template for the first PCR amplification and 1 μl of the obtained amplicons as a template for a second semi-nested PCR, which was conducted with tagged primers. Tags were designed as described in Fiore-Donno et al. (2018). The used primers and tag combinations are provided in Supplementary Tables 3, 5.

We applied the following final concentrations: DreamTaq polymerase (Thermo Fisher Scientific, Dreieich, Germany) 0.01 units, Thermo Scientific DreamTaq Green Buffer, dNTPs 0.2 mM and primers 1 μM. To reduce the artificial dominance of few amplicons by PCR competition, all PCRs were carried out twice. At least two negative controls were included for every PCR to rule out possible cross-contaminations. PCR products were pooled, then purified and normalized using SequalPrep Normalization Plate Kit (Invitrogen GmbH, Karlsruhe, Germany). Sequencing was performed with a MiSeq v2 Reagent kit of 500 cycles for the shorter ITS amplicons (c. 250 bp) of Oomycota and a MiSeq v3 Reagent kit of 600 cycles for the amplified V4 Region fragments (c. 350 bp) of Cercozoa. Sequencing was conducted by a MiSeq Desktop Sequencer (Illumina Inc., San Diego, CA, United States) at the Cologne Center for Genomics (Germany).

### Sequence Processing

All bioinformatic and statistical methods were applied to both Oomycota and Cercozoa datasets independently if not stated otherwise. Raw reads were merged using VSEARCH v2.10.3 (Rognes et al., 2016) at default settings. Merged contigs were demultiplexed with cutadapt v1.18 (Martin, 2011) allowing no mismatches in neither primer nor tag sequence. Cutadapt was also used to trim primer and tag sequences after demultiplexing. Sequences were then *de novo* clustered into operational taxonomic units (OTUs) using Swarm v2.2.2 (Mahé et al., 2015) with *d* = 1 and fastidious option on, i.e., sequences differing in one nucleotide were added to the cluster. Chimeras were *de novo* detected using VSEARCH. OTUs were removed from the final OTU table if they were flagged as chimeric, showed a quality value of less than 0.0002, were shorter than 150 bp (Oomycota) or 300 bp (Cercozoa), or were represented by less than 0.005% of all reads (Nelson et al., 2014; Sapp et al., 2018; i.e., 141 reads for Oomycota or 269 reads for Cercozoa).

For taxonomic assignment, OTUs were first tentatively assigned by using BLAST+ v2.9.0 (Camacho et al., 2009) with default parameters against the non-redundant NCBI Nucleotide database (as of June 2019). OTUs were removed if the best hit in terms of bitscore was a non-oomycete or non-cercozoan sequence, respectively. For a finer taxonomic assignment, two databases were used: The PR2 database (v4.12.0, Guillou et al., 2012) served as a taxonomic reference set for cercozoan V4 sequences, while for the Oomycota all available oomycete sequences were downloaded from NCBI Nucleotide (as of July 2019). Both databases were used as a template for an *in silico* PCR with cutadapt, with the same primer sequences used in this study. The resulting virtual amplicons served as a database with the same length and genetic origin as our sequenced amplicons, which offers the advantage of penalizing terminal gaps during the taxonomic annotation – which was performed with VSEARCH. The annotation was refined by assigning the species name of the best VSEARCH hit to the corresponding OTU if the pairwise identity was over 95%. OTUs with lower percentages were assigned higher taxonomic levels.

To account for random effects due to low sequencing depth, the final OTU table was loaded into QIIME 2 v2018.11 (Bolyen et al., 2019) to explore the sequencing depth by sample metadata. The minimum sequencing depth was determined depending on how many samples per metadata would be excluded. It was set as high as possible, while retaining at least five samples per microhabitat and 15 samples per tree species and resulted in a minimum sequencing depth of 9,588 sequences for oomycete samples and 15,684 sequences for cercozoan samples.

### Statistical Analyses

All statistical analyses were conducted in R v3.5.3 (R Core Team, 2019). Rarefaction curves were carried out with the iNEXT package (Chao et al., 2014; Hsieh et al., 2019) to determine if a higher sequencing depth would have revealed more OTUs. Alpha diversity indices were calculated for each sample using the *diversity* function in the vegan package (Oksanen et al., 2019) and pairwise significance was tested with Tukey’s Honest Differences (function *HSD.test*) as implemented in the agricolae package (De Mendiburu and Yaseen, 2020). The former methods were applied on the OTU table with absolute abundances. To explore differences in the community composition across the samples, the following beta diversity-based methods were conducted on relative abundances. Non-metric multidimensional scaling (NMDS) was performed on the Bray-Curtis dissimilarity matrix of the log transformed table (functions *vegdist* and *metaMDS* in the vegan package, respectively). The same method was used in a permutational multivariate analysis of variance (permANOVA, function *adonis*), to test if oomycete and cercozoan OTU diversity differed across the strata, habitats and tree species. To analyze the effects of environmental factors to the variance of the community composition, a redundancy analysis was carried out on the Hellinger-transformed table (function *rda* in the vegan package). The function *nestedtemp* (vegan package) was used to test if communities or patches of species poor microhabitats might be a subset of species rich communities. Species accumulation curves were calculated using the *specaccum* function and the number of shared OTUs between different combinations of microhabitats was visualized using the UpSetR package (Lex et al., 2014; Gehlenborg, 2019). An indicative value analysis (Dufrêne and Legendre, 1997) was performed with the indicspecies package (De Cáceres and Legendre, 2009) to identify indicator taxa in the different microhabitats. All figures were plotted with the ggplot2 package (Wickham, 2016).

## Results

### Sequencing and Bioinformatic Pipeline

**Figure 1:**
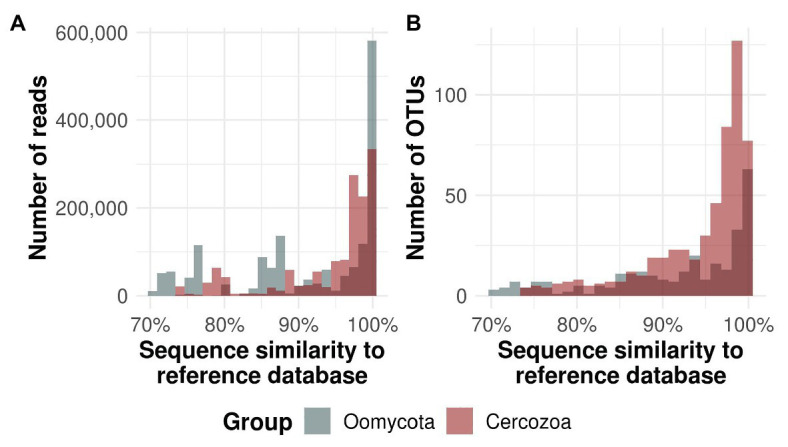
Similarity of protistan sequences to the taxonomic reference database. Oomycete sequences and operational taxonomic units (OTUs) are given in gray bars, cercozoan in red. Dark color represents the overlap between the bars. The majority of all reads **(A)** and OTUs **(B)** were ≥97% similar to the respective database. Around 17.2% of all oomycete OTUs had <70% similarity to known reference sequences, whereas only 0.5% of the cercozoan reads had a similarity of <70% (not shown).

We obtained 550 OTUs from 1.381.839 sequences (Cercozoa) and 331 OTUs from 1.610.374 sequences (Oomycota). The average number of cercozoan OTUs was 516 ± 15 and 546 ± 3 per microhabitat and tree species, respectively, while the average number of oomycete OTUs was 236 ± 25 and 304 ± 4 per microhabitat and tree species, respectively. Tree canopies contained a substantially unknown diversity of oomycetes, with 57 oomycete OTUs with less than 70% percent identity to any known reference sequence, while this was the case for only three cercozoan OTUs (Figure 1). While most of the reads and OTUs showed a similarity of 97–100% to any known reference sequence, oomycete reads revealed two additional peaks at about 76 and 87%, indicating that a small but significant number of highly abundant OTUs in oomycetes is still not taxonomically recorded (see Supplementary Figure 1 and Supplementary Tables 1, 2 for taxonomic composition and annotation).

### Alpha Diversity

**Figure 2:**
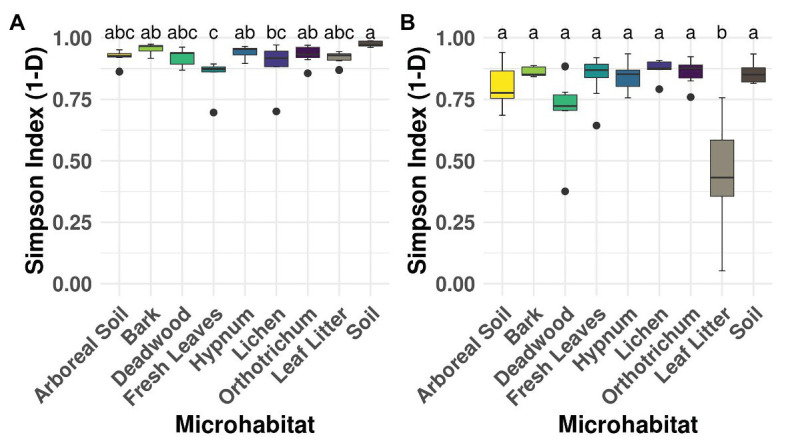
Alpha diversity of microhabitats for cercozoan **(A)** and oomycete **(B)** communities. Boxplots describe the Simpson Index of the samples grouped by microhabitat; outliers are given by dots. Letters correspond to Tukey’s Honest Difference *post hoc* test, with microhabitats not sharing any letter having significantly different means. Simpson Index revealed high alpha diversity irrespective of the investigated protistan group, with the exception of lower alpha diversity of the leaf litter samples within the Oomycota.

The used taxon-specific primers thoroughly recovered the OTU richness of canopy and ground samples (soil and litter) as indicated by rarefaction curves (Supplementary Figure 2). The extrapolation showed that doubling the sequencing depth would have yielded no more cercozoan or oomycete OTUs. All sampled microhabitats showed high alpha diversity (Figure 2), except for oomycetes in the ground leaf litter (ANOVA *F* value = 10.79, *p* < 0.001; Figure 2B).

### Beta Diversity

**Figure 3:**
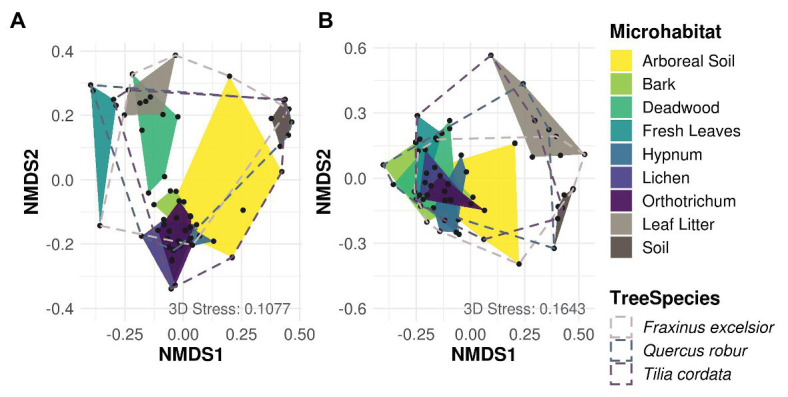
Non-metric multidimensional scaling (NMDS) of Bray-Curtis dissimilarities of cercozoan **(A)** and oomycete **(B)** communities among microhabitats. Cercozoan communities showed a finer separation between canopy microhabitats compared to Oomycota, while the latter showed a clearer separation of communities between canopy (green and yellow) and ground (brown). Stress values of NMDS are shown in the lower right of each graph. Microhabitats were more influential for protistan community composition than tree species (Supplementary Table 5).

Most variation in Cercozoa and Oomycota beta diversity was explained by microhabitat differences (permANOVA, Cercozoa: *R*^2^ 0.45, *p* = 0.001; Oomycota: *R*^2^ 0.30, *p* = 0.001). In addition, the beta diversity of oomycetes differed between tree species with a small, but significant proportion of explained variance, but not beta diversity of Cercozoa (permANOVA; Cercozoa: *R*^2^ 0.04, *p* = 0.1; Oomycota: *R*^2^ 0.06, *p* = 0.01; Supplementary Table 5). NMDS reflected a strong separation of community profiles between canopy and ground strata (Figure 3; permANOVA; Cercozoa: *R*^2^ 0.13, *p* = 0.001; Oomycota: *R*^2^ 0.14, *p* = 0.001), showing that the composition of soil and litter communities was thoroughly different from the canopy inhabiting communities, especially in oomycetes. In Cercozoa, communities inhabiting fresh canopy leaves were most distinct to those in mineral soil on the ground (Figure 3A). However, cercozoan leaf litter communities were more similar to communities detected on fresh canopy leaves than to the underlying soil communities. A clear difference of beta diversity between cercozoan communities of bark and epiphytes with lichen and mosses (*Hypnum* sp. and *Orthotrichum* sp.) could not be observed, but they were clearly distinct to communities of deadwood and to those of fresh canopy leaves (Supplementary Table 6). Communities of arboreal soil were highly variable, ranging from samples with high similarity to mosses to samples closely resembling the mineral soil communities underneath the litter layer on the ground. Also, in Oomycota (Figure 3B), canopy, and ground communities were most different. Again, communities of the two ground samples, mineral soil and leaf litter, showed no overlap (Figure 3B), with leaf litter having a low alpha diversity (Figure 2). Oomycete communities of the different canopy microhabitats showed a high overlap and accordingly showed less microhabitat specificity than cercozoan communities (Figure 3B). Further, oomycete communities of fresh canopy leaves were clearly distinct from those of ground litter, which scaled closer to communities detected in soil samples. A shared pattern between cercozoan and oomycete communities in the canopy was their high variability of beta diversity in the arboreal soil.

A db-RDA showed the same pattern as NMDS but revealed more clearly the similarity of communities of fresh canopy leaves to deadwood in both protistan taxa (Supplementary Figure 3). For the Cercozoa (Supplementary Figure 3A), the first and second axis explained 15.4 and 12.8% of variance, respectively. The y-axis explained mostly the difference of cercozoan communities in mineral soil, while the x-axis explained the difference between communities in litter and deadwood in comparison to bark and epiphytes. Communities of fresh canopy leaves and litter on the ground were rather similar, and again bark and epiphyte communities did not differ. No clear pattern was found between tree species and arboreal soil. In the Oomycota, the x-axis explained 13.6% of variance and separated the canopy communities from communities of litter and mineral soil on the ground (Supplementary Figure 3B). Bark and epiphytes communities were similar and little separated along the y-axis from canopy leaf communities. Further, oomycetes of *Quercus* and *Fraxinus* were separated along the y-axis, which however explained only 5.6% of variance.

### Taxonomic Diversity

**Figure 4:**
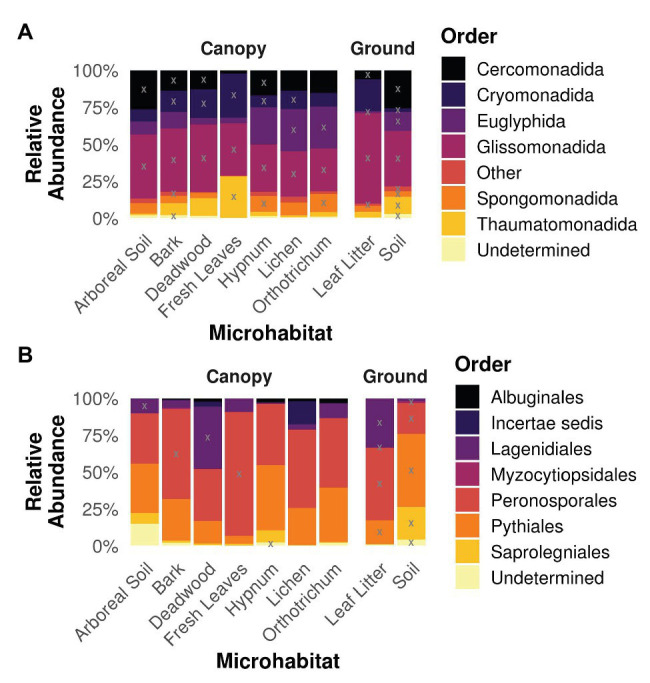
Taxonomy of Cercozoa **(A)** and Oomycota **(B)** partitioned on the different microhabitats. Gray “X”s represents indicative taxa within the respective order and microhabitat. Orders represented by less than 1% of all reads were concatenated to “Other” for the sake of clarity. Most cercozoan OTUs were assigned to Cercomonadida, Cryomonadida, Euglyphida, and Glissomonadida in all habitats, while oomycete habitats were dominated by Peronosporales and Pythiales.

Analysis of the taxonomy within the different microhabitats revealed similar patterns for both strata ground and canopy (Figure 4). The cercozoan order Glissomonadida dominated all habitats. With 40 ± 10.1% relative abundance per microhabitat followed by the orders Cercomonadida (14.8 ± 7.8%), Cryomonadida (13.8 ± 8.4%), and Euglyphida (13.8 ± 10.9%). The indicative value analysis determined OTUs belonging to these orders to be indicators for most habitats, while additionally OTUs from the Thaumatomonadida were indicative for fresh leaves and soil habitats and members of the Spongomonadida for the two mosses Hypnum and Orthotrichum as well as soil (Figure 4A). Oomycete communities were dominated by OTUs belonging to the orders Peronosporales (47.4 ± 18.2%) and Pythiales (28.3 ± 14.4%), with a high relative abundance of Lagenidiales in deadwood and leaf litter samples (42.3 and 33.3%, respectively; Figure 4B). OTUs belonging to the latter order were also determined to be indicative for these habitats. Additionally, OTUs from the Peronosporales were indicators for bark, leaf litter, and soil samples, and members of the Pythiales were indicative for deadwood, leaf litter, and soil samples.

### Shared OTUs

**Figure 5:**
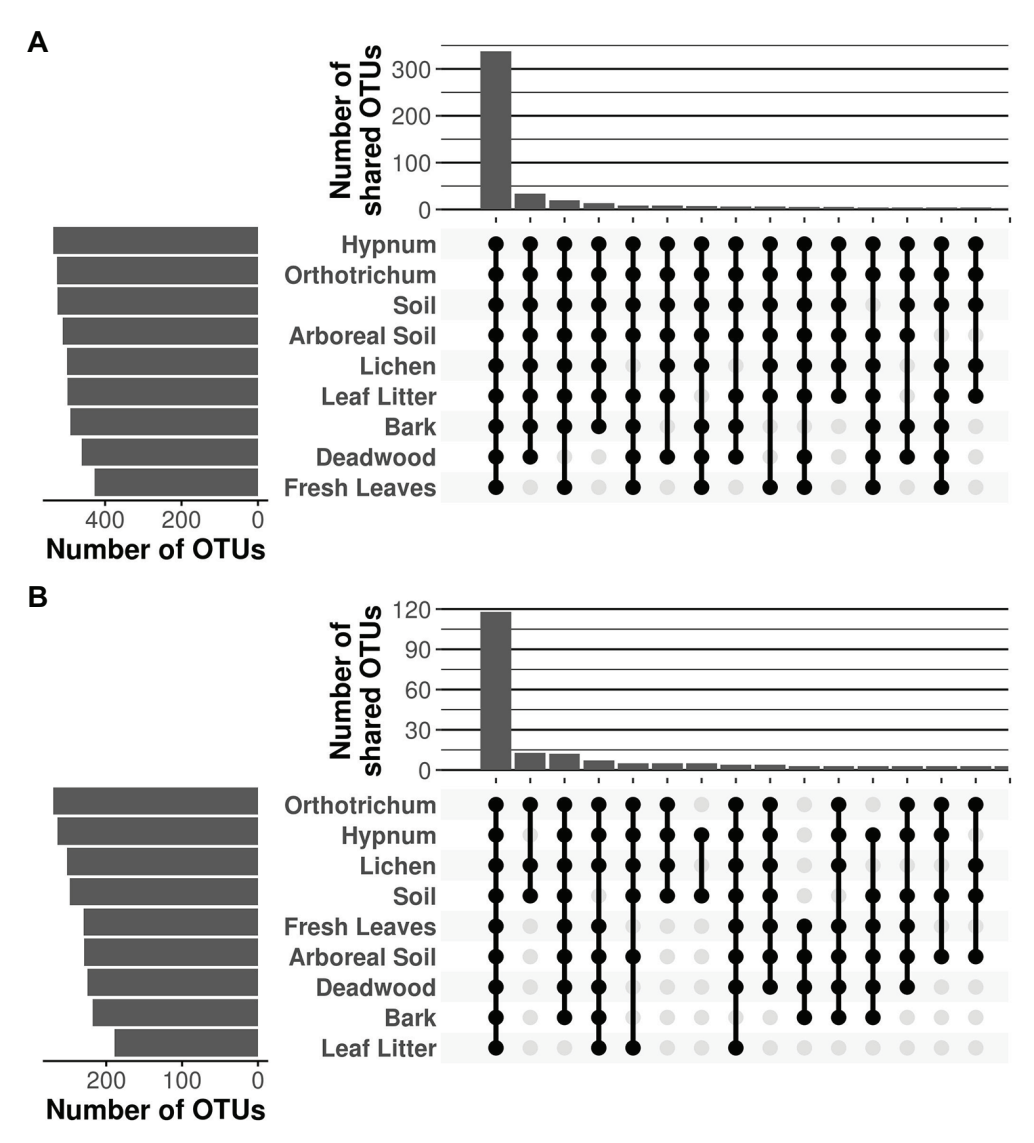
Shared OTUs of Cercozoa **(A)** and Oomycota **(B)** between microhabitats. Top bar chart represents the sum of the number of shared OTUs resulting from the combination of microhabitats in the matrix below. Only the 15 combinations with the highest numbers of shared OTUs are shown. The majority of OTUs were shared between all microhabitats, irrespective of the investigated protistan phylum.

Despite major differences in beta diversity, the majority of OTUs were shared between all microhabitats irrespective of the protistan phylum (Figure 5). Only a few combinations yielded more than 10 unique OTUs shared between distinct microhabitats, which is negligible given the high OTU richness per sampled microhabitat, which varied between 498 (deadwood) and 537 OTUs (fresh leaves) for the Cercozoa (Figure 5A) and between 189 (leaf litter) and 270 (*Orthotrichum* moss) for the Oomycota, respectively (Figure 5B). Because almost all OTUs were shared between all microhabitats with the species accumulation curve showing only a flat increase, communities revealed no patterns of nestedness (Supplementary Figures 4, 5).

## Discussion

Application of taxon-specific primers ensured an exhaustive coverage of the investigated protistan taxa, which has two major consequences: First, OTU richness of both Cercozoa and Oomycota in our study is at least an order of magnitude higher than in studies using general eukaryotic primers (Lentendu et al., 2014; Geisen et al., 2015; Mahé et al., 2017). Thus, the majority of detected OTUs account for a large undescribed diversity (Figure 1) and may represent so far uncharacterized lineages, especially in the phylum Oomycota, as only 34% of the OTUs were 97–100% similar to any known sequenced species. Second, the majority of all OTUs were detected in all microhabitats, a crucial precondition to avoid an erroneous classification of species as absent or rare by undersampling (Supplementary Figure 2).

Our data show how discrete microhabitat niches lead to compositional heterogeneity of microbial communities in tree canopies and ultimately within entire tree-based ecosystems (Peay et al., 2016; Cregger et al., 2018). Beta diversity of canopy communities was strikingly different to mineral soil communities on the ground (Figure 3A). In addition, different microhabitats within tree canopies were further colonized by distinct cercozoan communities. However, we could not confirm an increased species richness with increasing habitat diversity. One explanation for this observation could be the dominance of generalist species with high dispersal capacity (Finlay et al., 2001; Bahram et al., 2016), which in turn might have led to observed consistent species richness. Thus, differences in beta diversity were solely driven by differences in relative abundance between protistan taxa due to better adaptations to habitat-specific conditions (i.e., habitat filtering). The ubiquitous distribution of Cercozoa and Oomycota shows that generally most taxa can occur everywhere (Figures 4, 5), but the occurrence of only a few specialist OTUs does not imply functional homogenization at the community level across microhabitats, but rather indicates that with increasing habitat diversity functional diversity could increase to a greater extent than OTU richness (Ofek-Lalzar et al., 2014).

Our findings differ from patterns observed for bacteria (Lundberg et al., 2012; Ottesen et al., 2013; Wagner et al., 2016), epifoliar fungi (Gilbert et al., 2007), or lichens (Boch et al., 2013; Marmor et al., 2013), where highly specialized microhabitat communities have been reported. Primary consumers such as bacteria experience a direct selective pressure by differences in resource composition between plant microhabitats (Vandenkoornhuyse et al., 2015; Thapa et al., 2017; Sasse et al., 2018). Compared to the highly specific bacterial communities of tree bark, mosses, and lichens (Aschenbrenner et al., 2017), canopy protists detected in our study appear to rather depend on general microhabitat characteristics than on a specific microhabitat identity. This is best exemplified within the cryptogamic epiphytes. Lichen and the two moss taxa harbored quite similar cercozoan and oomycete communities (Figure 3). These epiphytes are characterized by quickly changing conditions with rapid swelling and storage of moisture from morning dew and after rainfall to severe dryness at sunshine (e.g., Jonsson et al., 2015; Benítez et al., 2018), which to a certain degree may act as environmental filters favoring specific protistan taxa. In contrast, cercozoan and oomycete communities in arboreal soil samples showed high variability in beta diversity, spanning from moss-like communities to soil-like communities (Figure 3). This indicates that observed communities resembling those of mineral soil are not restricted to the forest floor. Thus, community variability in arboreal soil might be due to the varying degree of decay of the sampled material and its distinct physicochemical properties (Nadkarni et al., 2002), which further supports our observation that increasing habitat heterogeneity results in increasing dominance of certain protistan taxa, which determine the compositional heterogeneity of cercozoan and oomycete communities.

Mahé et al. (2017) hypothesized that soil protists could also be found in the canopy from where they might have been washed down; a pattern confirmed for leaf endophytic fungi in 1-year old beech litter of temperate forests (Guerreiro et al., 2018). Also, cercozoan communities were surprisingly similar between canopy leaves and leaf litter on the ground (Figure 3A), suggesting that the phyllosphere may substantially contribute to community assembly of cercozoan litter communities. A growing number of studies lend support to this hypothesis by identifying particular cercozoan species predominantly adapted to life in the phyllosphere (Dumack et al., 2017; Flues et al., 2017; Öztoprak et al., 2020). Oomycetes on the other hand showed significantly different patterns of beta diversity between phyllosphere and ground litter, showing that this pattern cannot be confirmed for protists in general and that different protistan groups do not behave uniformly. While fresh canopy leaves and ground litter had highest OTU richness of Cercozoa (Figure 5A), ground litter contained a significantly depleted diversity of Oomycota (Figure 2B). The small, but significant differences of oomycete communities between tree species (Supplementary Figure 3B; Supplementary Table 5) might be explained by differences in host specificity, since oomycetes are well known to contain specific pathogens infecting leaves, stems, and roots of forest trees (e.g., Rizzo and Garbelotto, 2003; Lehtijärvi et al., 2017), which deserve further attention in future studies.

The ubiquity of the OTUs is also reflected on a taxonomic scale (Figure 4). Cercozoans belonging to the class of Sarcomonadea (Glissomonadida and Cercomonadida) have been shown to dominate various terrestrial habitats (Geisen et al., 2015; Ploch et al., 2016; Fiore-Donno et al., 2018). Aforementioned pattern was also observed in the investigated canopy and ground samples, further indicating a high dispersal rate and habitat generalists within these orders. However, indicative value analysis determined OTUs belonging to these orders to be indicators for most habitats, thus the presence of specialist OTUs is not negligible. Most oomycete OTUs were assigned to the Peronosporales and Pythiales, irrespective of the microhabitat (Figure 4B). This dominance is not surprising as Peronosporales and Pythiales comprise the highest number of described oomycete species (Thines, 2014). A prominent exception however is the dominance of Lagenidiales within deadwood and leaf litter communities. Members of the Lagenidiales have hitherto been described as obligate parasites for aquatic organisms (Sparks, 1985; Nakamura and Hatai, 1995) and mammals, including humans (Mendoza and Vilela, 2013). The Lagenidiales in our study, however, only show a pairwise identity of 76% or less to any known reference sequence, which therefore might represent undescribed lineages independent from lifestyles described for known Lagenidiales.

## Conclusion

Beta diversity of Cercozoa and Oomycota was solely driven by differences in the relative abundance of OTUs, because almost all taxa did occur ubiquitously among tree crowns and soil of the floodplain forest. Accordingly, species richness did not increase with habitat diversity as hypothesized and the strong differences in beta diversity between protistan communities of the forest floor and tree crowns and among microhabitats within tree crowns can be almost solely attributed to differences in relative abundance. Taxonomic differences between tree species had a surprisingly low influence on cercozoan community assembly; even the mostly plant-parasitic oomycetes did not show a high degree of host-specificity. Being mainly secondary consumers, the low host specificity of both investigated protistan taxa appears as a major difference to the often-high host specificity of microbial primary consumers. Both strata, forest floor and canopy showed quite unique cercozoan and oomycete communities, but communities of arboreal soil became more similar to those in mineral soil. Cercozoan communities of canopy leaves differed little from Cercozoa in the litter layer on the ground, indicating strong selective forces of microhabitat conditions independent of the canopy or ground stratum. Thus, our findings indicate that the diversity of terrestrial protists is strongly shaped by habitat filtering, but – a thorough taxon sampling provided – species richness is hardly affected.

## Data Availability Statement

The datasets presented in this study can be found in online repositories. The names of the repository/repositories and accession number(s) can be found below: https://www.ebi.ac.uk/ena, ERS4399743, https://www.ebi.ac.uk/ena, ERS4399744. Code and detailed bioinformatic/statisticalmethods used in this study are available at: https://github.com/RJauss/FromForestSoilToCanopy.

## Author Contributions

MB and MS designed the study. RW and SS conceived and conducted the sampling and DNA extraction. AF-D contributed the primers, KD helped with the laboratory work. SW and R-TJ conducted the PCRs, performed the bioinformatic and statistical analyses and drafted the manuscript. All authors contributed to the article and approved the submitted version.

### Conflict of Interest

The authors declare that the research was conducted in the absence of any commercial or financial relationships that could be construed as a potential conflict of interest.
