# Supplementary material for: From Forest Soil to the Canopy: Increased Habitat Diversity Does Not Increase Species Richness of Cercozoa and Oomycota in Tree Canopies
Source: Front Microbiol. 2020 Dec 22;11:592189. doi: 10.3389/fmicb.2020.592189 (PMC7782269; doi:10.3389/fmicb.2020.592189)
Supplement: Supplementary file 2 [file Data_Sheet_2.pdf]

Supplementary Figures

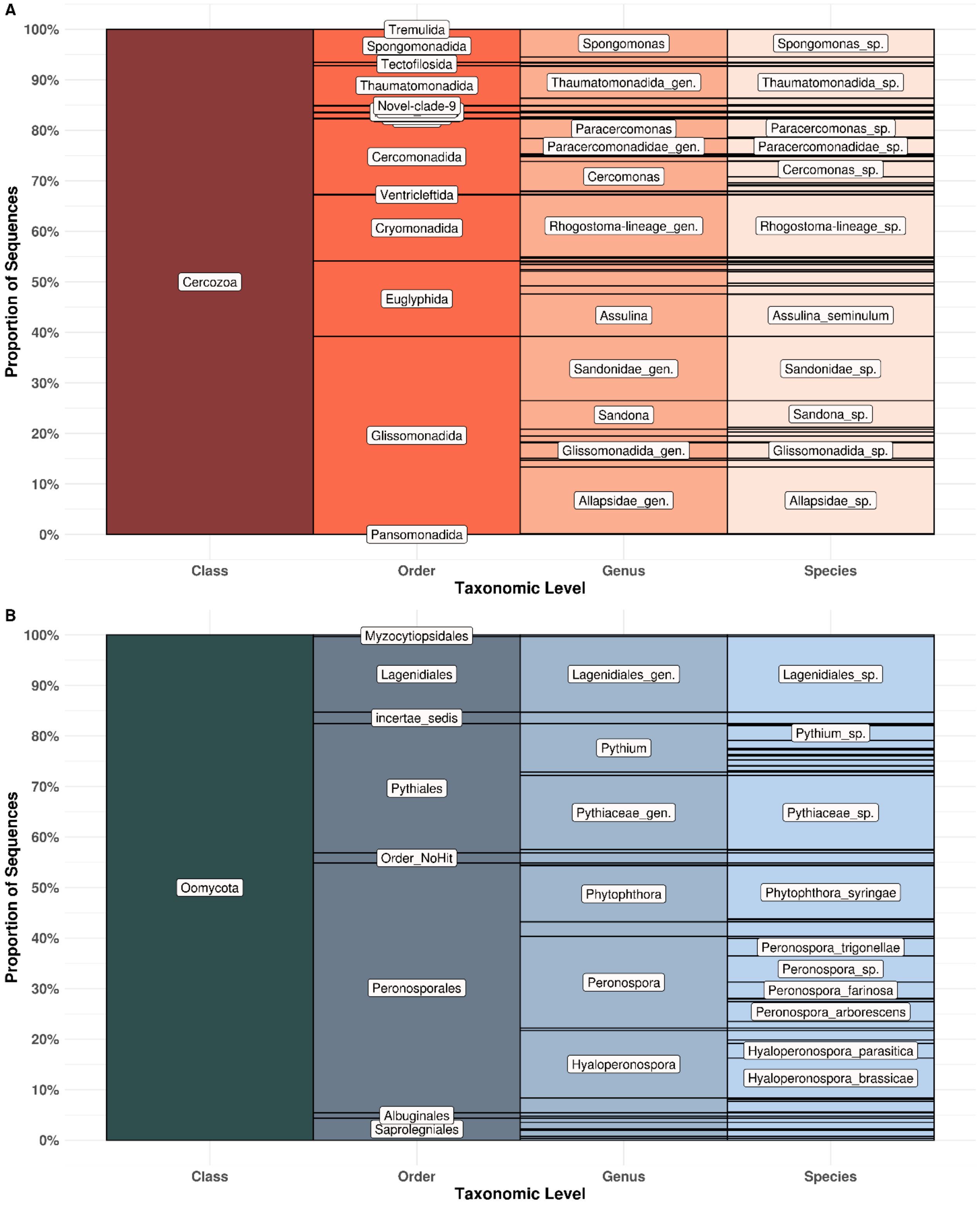

Supplementary Figure 1. Taxonomic composition of cercozoan (A) and oomycete (B) sequences. Labels give the detected orders and the ten most abundant species with their corresponding genus.

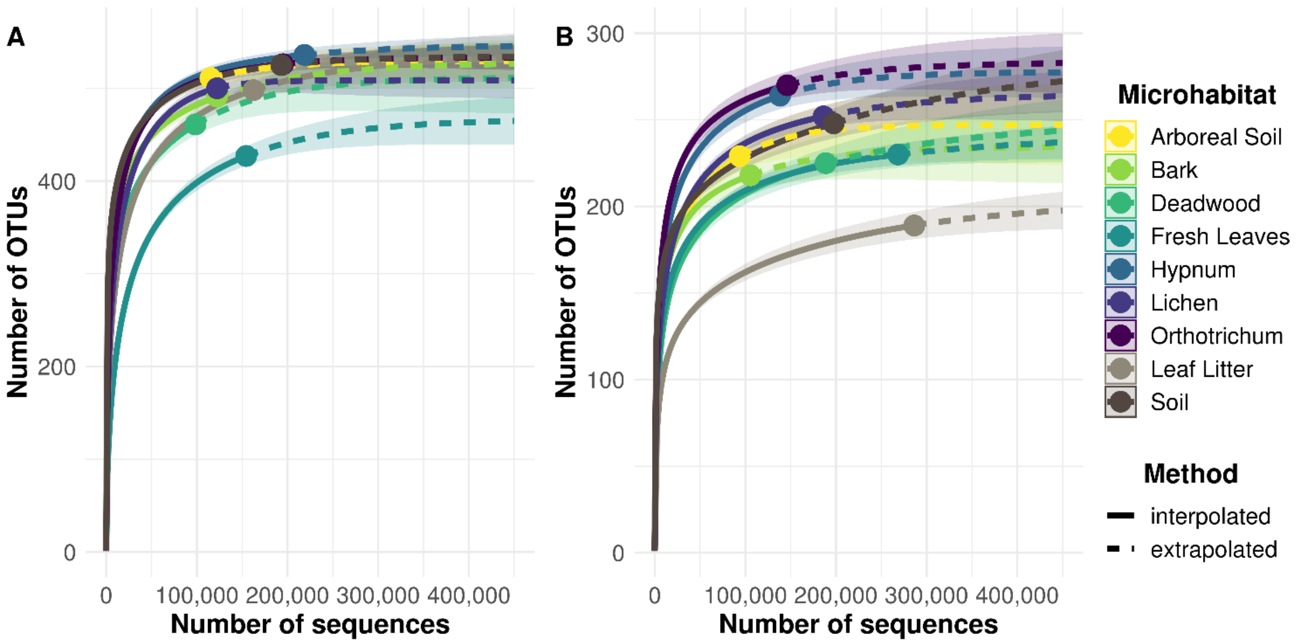

Supplementary Figure 2. Rarefaction curves of cercozoan (A) and oomycete (B) datasets. Solid lines give the interpolated number of OTUs from subsampled sequences, dashed lines represent extrapolated number of OTUs with increasing number of sequences. Shaded areas give the 97% confidence intervals.

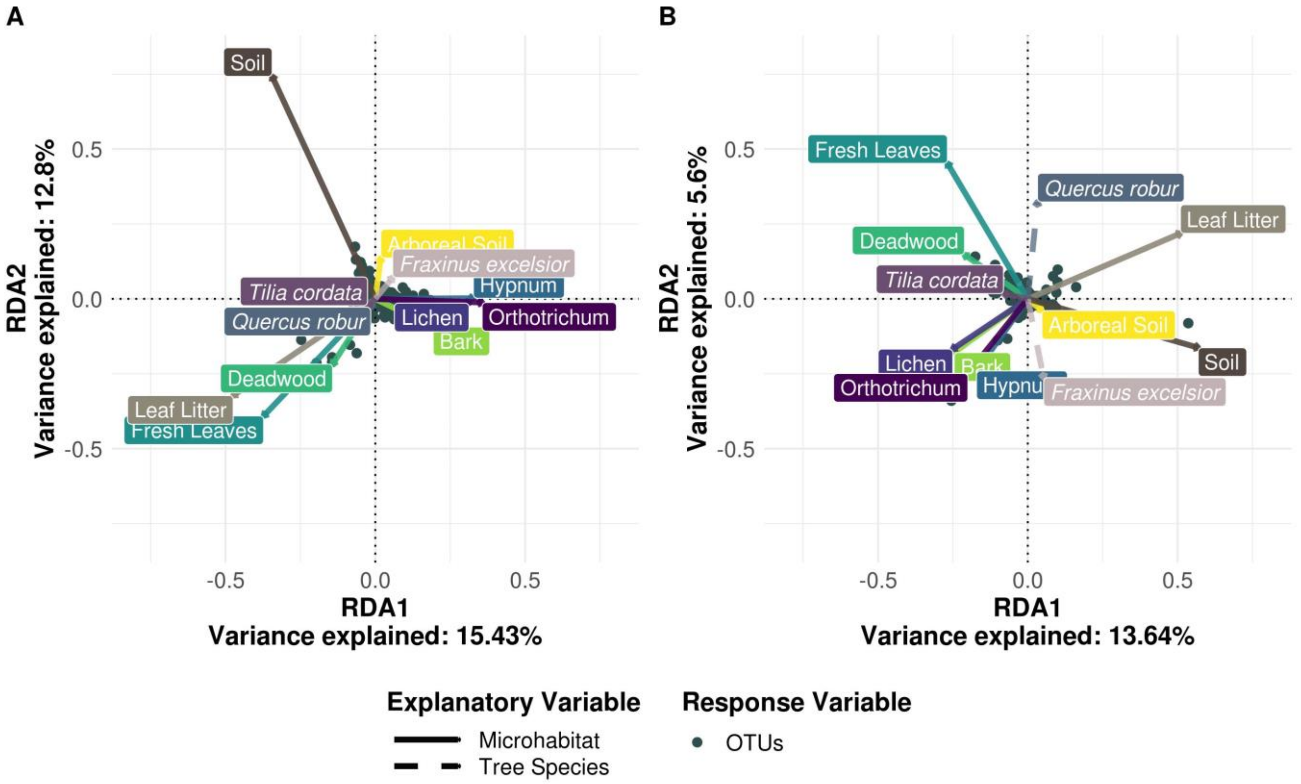

Supplementary Figure 3. Redundancy analysis (db-RDA) of cercozoan (A) and oomycete (B) OTUs, microhabitats and tree species. Environmental factors of microhabitat identity and tree species were included in the analysis. Dots represent OTUs. The percentages of variability explained by each axis (RDA1 and RDA2) are given in the labels. RDA ordination resulted in three clusters explaining a comparable amount of variance respectively.

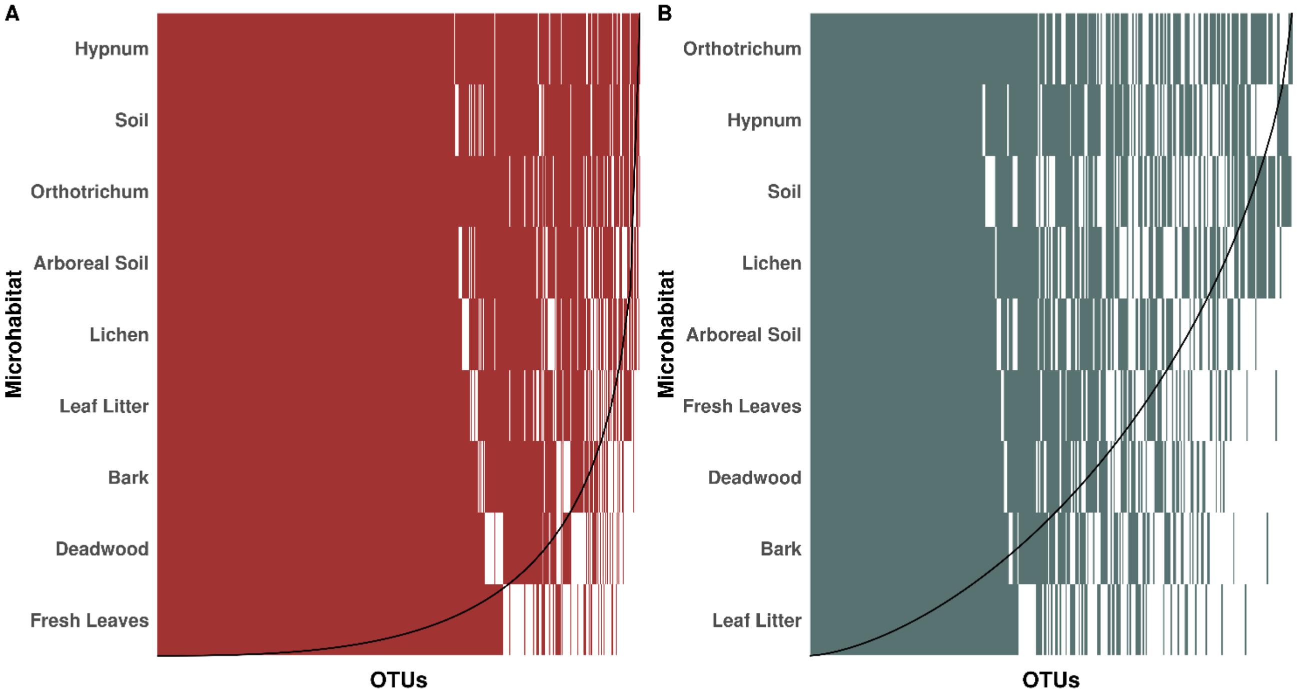

Supplementary Figure 4. Nestedness analysis of cercozoan (A) and oomycete (B) datasets. Each coloured bar in the incidence matrix gives the presence of the respective OTU in the microhabitat, blank fields the absence. The solid black line represents the pattern which would be observed in a perfectly nested dataset. The observed distribution of OTU incidence show no pattern of nestedness.

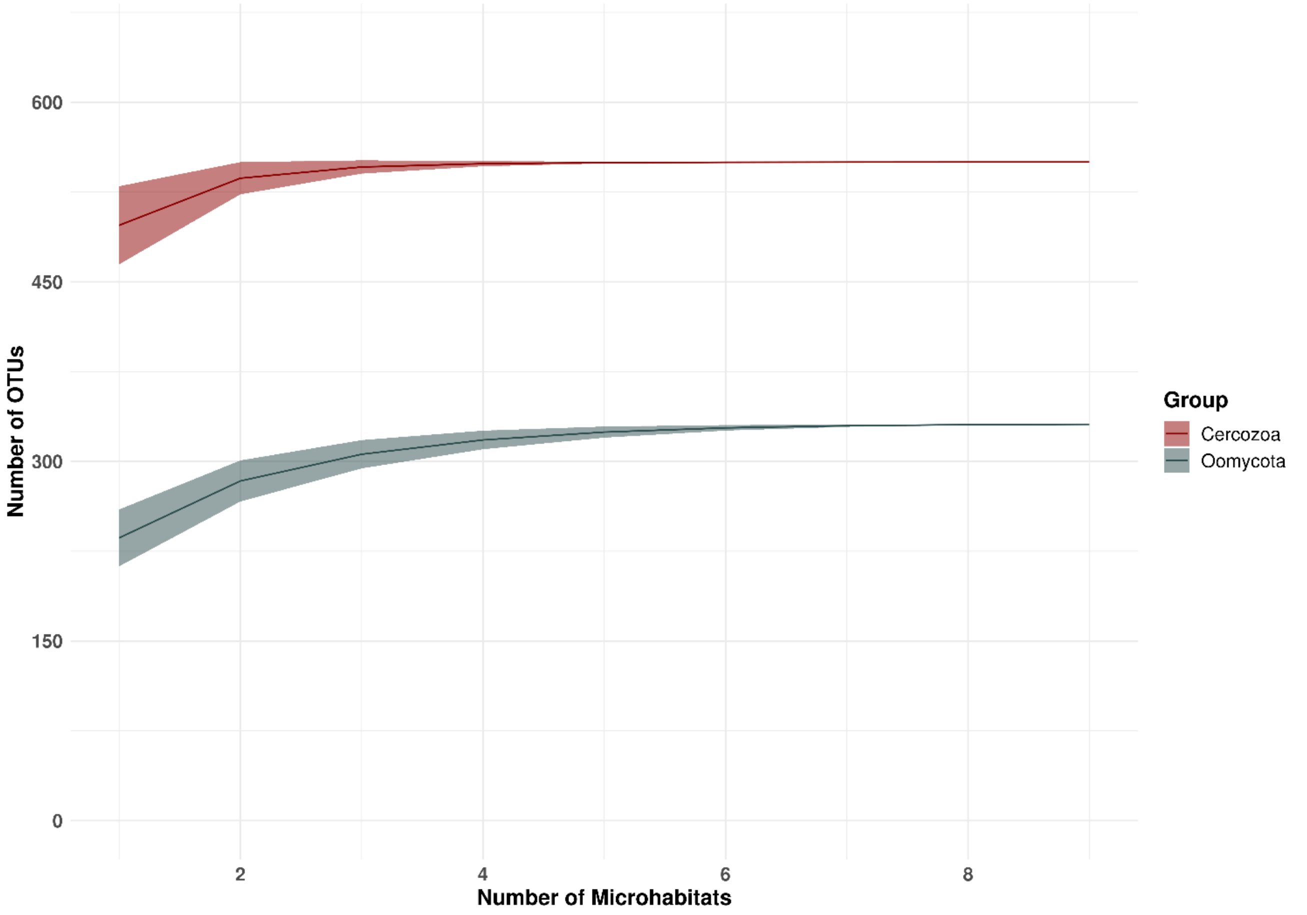

Supplementary Figure 5. Species accumulation curves of cercozoan (red) and oomycete (grey) datasets. Solid lines represent the increase in species richness with increasing number of sampled microhabitats. Shaded areas give the standard deviation. As the curves reach a plateau with only few samples, it can be assumed that nearly all OTUs can be detected by sampling only few microhabitats.
